# Protein‐Induced Pluripotent Stem Cells Ameliorate Cognitive Dysfunction and Reduce Aβ Deposition in a Mouse Model of Alzheimer's Disease

**DOI:** 10.5966/sctm.2016-0081

**Published:** 2016-08-15

**Authors:** Moon‐Yong Cha, Yoo‐Wook Kwon, Hyo‐Suk Ahn, Hyobin Jeong, Yong Yook Lee, Minho Moon, Sung Hoon Baik, Dong Kyu Kim, Hyundong Song, Eugene C. Yi, Daehee Hwang, Hyo‐Soo Kim, Inhee Mook‐Jung

**Affiliations:** ^1^ Department of Biochemistry and Biomedical Sciences, Seoul National University, Seoul, Republic of Korea; ^2^ Innovative Research Institute for Cell Therapy, Seoul National University Hospital, Seoul, Republic of Korea; ^3^ National Research Laboratory for Stem Cell Niche, Seoul National University, Seoul, Republic of Korea; ^4^ Department of New Biology and Center for Plant and Aging Research, Institute for Basic Science, Daegu Gyeongbuk Institute of Science and Technology, Daegu, Republic of Korea; ^5^ The Korean Ginseng Research Institute, Daejeon, Republic of Korea; ^6^ Department of Biochemistry, College of Medicine, Konyang University, Daejeon, Republic of Korea; ^7^ Department of Molecular Medicine and Biopharmaceutical Sciences, Graduate School of Convergence Science and Technology, School of Medicine and School of Pharmacy, Seoul National University, Seoul, Republic of Korea

**Keywords:** Alzheimer's disease, Protein‐iPSC, 5XFAD mice, Proteomic analysis, Oligodendrocyte

## Abstract

Transplantation of stem cells into the brain attenuates functional deficits in the central nervous system via cell replacement, the release of specific neurotransmitters, and the production of neurotrophic factors. To identify patient‐specific and safe stem cells for treating Alzheimer's disease (AD), we generated induced pluripotent stem cells (iPSCs) derived from mouse skin fibroblasts by treating protein extracts of embryonic stem cells. These reprogrammed cells were pluripotent but nontumorigenic. Here, we report that protein‐iPSCs differentiated into glial cells and decreased plaque depositions in the 5XFAD transgenic AD mouse model. We also found that transplanted protein‐iPSCs mitigated the cognitive dysfunction observed in these mice. Proteomic analysis revealed that oligodendrocyte‐related genes were upregulated in brains injected with protein‐iPSCs, providing new insights into the potential function of protein‐iPSCs. Taken together, our data indicate that protein‐iPSCs might be a promising therapeutic approach for AD. Stem Cells Translational Medicine *2017;6:293–305*

**Significance Statement**

Alzheimer's disease (AD) leads to cognitive dysfunctions without methods for cure or prevention. Here we demonstrated that transplantation of protein‐induced pluripotent stem cells (iPSCs) reduced plaque deposition and restored memory impairment in 5XFAD mice, an AD animal model. Also, the stem cell niche of these mice promotes differentiation of protein‐iPSCs to glial cells, especially oligodendrocytes. Comparative analysis of the proteome revealed that oligodendrocyte‐related genes, including transferrin, were upregulated in the transplanted stem cell niche and that transferrin enhanced differentiation of protein‐iPSCs into oligodendrocytes by promoting its maturation. Therefore, protein‐iPSCs may provide potential therapeutic interventions with a glial cell‐derived approach for neurodegenerative disorders, including AD.

## Introduction

Alzheimer's disease (AD) is one of the most prevalent neurodegenerative diseases that lead to cognitive dysfunction 1, 2. Although the molecular mechanisms for its pathophysiology are not clearly understood, many studies have reported that soluble Aβ, or amyloid, plaques in the brain are causative factors leading to neuronal loss and memory impairment 3 4 5. Recently, the importance of glial cells, such as astrocytes, microglia, and oligodendrocytes, in the ontogeny of neurodegenerative diseases, including AD, has been identified 6 7 8 9. Glial cells are also involved in the release of neuromodulatory factors that influence neuronal activity and intracellular signaling 10, 11. These findings indicate that restoring not only damaged neurons but also dysfunctional glial cells may be crucial for the development of an AD therapeutic strategy 12 13 14.

In line with these observations, pluripotent stem cells are a promising regenerative medicine because they can be differentiated to various cell types in the central nervous system 15. It has been reported that AD pathogenesis and memory impairment can be mitigated by transplanting mesenchymal stem cells into an AD animal model 16 17 18 19 20. Previously, we found that the delivery of whole embryonic stem cell (ESC) extracts into adult fibroblasts enables them to convert pluripotent stem cells (iPSCs) without transduction of defined factors 21. During the reprogramming process, global gene expression and epigenetic status are converted from somatic to ES cell‐equivalent status 21. These iPSCs (called protein‐iPSCs) are indistinguishable from ESCs in their potential for differentiation. These cells differentiate into all three germ layers: the endoderm, mesoderm, and ectoderm 21. Furthermore, these cells show complete developmental potency, and all chimeric mice developed from protein‐iPSCs have lived for more than 40 weeks in good condition without any tumors 21. This implies that our protein‐iPSCs are able to overcome the safety issues associated with genetically modified iPSCs. In the present study, we examined whether protein‐iPSC transplantation into the brain of an AD mouse model has the potential to treat AD and, if so, how they would render a beneficial effect on AD pathogenesis.

## Materials and Methods

### Generating and Maintaining Mouse Protein‐iPSCs

Protein‐iPSCs were cultured as described elsewhere 21, 22. The C57BL/6‐background mouse ESCs (C57‐mESCs, accession no. SCRC‐1002; American Type Culture Collection, Manassas, VA, https://www.atcc.org/) were cultured on mitomycin C (Sigma‐Aldrich, St. Louis, MO, http://www.sigmaaldrich.com/)‐treated SIM mouse embryo‐derived thioguanine‐ and ouabain‐resistant (STO) feeder layer in 0.1% gelatin (Sigma‐Aldrich)‐coated tissue culture dish 23, 24. STO cells were cultured in Dulbecco's modified Eagle's medium (DMEM; Thermo Fisher Scientific Life Sciences, Waltham, MA, http://www.thermofisher.com) high glucose supplemented with 10% fetal bovine serum (FBS; Thermo Fisher), 100 U/ml penicillin streptomycin (Thermo Fisher). To induce the reprogramming of adult fibroblasts, ESC‐derived extract proteins were prepared and transferred using streptolysin O (Sigma‐Aldrich)‐mediated reversible permeabilization. Twenty to 35 mg/ml protein was used to induce reprogramming. When primary colonies were observed, they were reseeded on an STO feeder layer and subcultured. One day before subculturing of protein‐iPSCs, mitomycin C (10 μg/ml medium, Sigma‐Aldrich)‐treated STO cells were seeded on a new 0.1% gelatin‐coated dish. Propagating protein‐iPSCs were cultured in DMEM with 10% defined FBS, 2 mM L‐glutamine (Thermo Fisher), 1× nonessential amino acids (Thermo Fisher), 1 mM 2‐mercaptoethanol (Sigma‐Aldrich), and 100 units/ml penicillin/100 μg/ml streptomycin (Thermo Fisher) embryonic stem cell media [ES media]). In ES media, 2000 U/ml of ESGRO leukemia inhibitory factor (EMD Millipore, Billerica, MA, https://www.emdmillipore.com) was added to maintain pluripotency. Protein‐iPSCs were dissociated with 0.05% trypsin (Thermo Fisher) and passaged on STO approximately every 2–3 days. Cells from passages 5–7 cells (culture days 45–55) were used for further experiments.

### Alkaline Phosphatase and Immunocytochemistry

Protein‐iPSCs were cultured as described previously 21, 22. Alkaline phosphatase (AP) staining was performed using an AP detection kit (BCIP/NBT, Promega, Madison, WI, http://www.promega.com). For immunocytochemical staining, protein‐iPSCs were fixed with 4% paraformaldehyde and blocked with 1% bovine serum albumin; in the case of nucleus cytoplasmic side staining, 0.1% Triton X‐100 was added. Staining was carried out by using primary anti‐Oct4 (1:100; Santa Cruz Biotechnology, Santa Cruz, CA, http://www.scbt.com/), anti‐Nanog (1:100; Santa Cruz Biotechnology), anti‐stage‐specific embryonic antigen 1 (SSEA1) (1:100; Santa Cruz Biotechnology), anti‐Nestin (1:100; Santa Cruz Biotechnology), anti‐Tra1 (1:100; Stemgent, Cambridge, MA, https://www.stemgent.com/), anti‐A2B5 (1:100; R&D Systems, Minneapolis, MN, https://www.rndsystems.com/), anti‐O4 (1:100; R&D Systems), anti‐myelin basic protein (MBP; 1:100; Abcam, Cambridge, U.K., http://www.abcam.com/), and samples were incubated overnight at 4°C. Appropriate Alexa Fluor dye conjugated secondary antibodies were as follows (all obtained from Thermo Fisher): donkey anti‐goat Alexa 555 (1:200), donkey anti‐mouse Alexa 488 (1:200), goat anti‐mouse IgM 488 (1:200), donkey anti‐rabbit Alexa 488 (1:200), goat anti‐mouse IgM Alexa 555 (1:200), and donkey anti‐mouse Alexa 488 (1:200). 4′,6‐Diamidino‐2‐phenylindole (1:2000, Thermo Fisher) was used for nuclear counterstaining. Images were obtained by using a confocal microscope (LSM 510 Meta; Carl Zeiss, Oberkochen, Germany, http://www.zeiss.com).

### Transgenic Mice

Transgenic mice with five familial AD mutations (5XFAD) used in this study were purchased from The Jackson Laboratory (Bar Harbor, ME). These mice express three mutations (Swedish: K670N, M671L; Florida: I716V; and London: V717I) of mutant human amyloid precursor protein (APP; 695) and human presenilin‐1, harboring two familial AD mutations (M146L, L286V). Animals were maintained in accordance with Animal Care and Use Guidelines of Seoul National University, Seoul, Republic of Korea.

### Stereotaxic Surgeries

Mice (4 months of age) were divided into four groups (15 mice per each group): wild type (WT) + saline, transgenic mice (Tg) + saline, Tg + ESCs, and Tg + protein‐iPSCs. Cells were tagged with fluorescent nanoparticle (Caladrius Biosciences, Basking Ridge, NJ, http://www.caladrius.com/) via endocytosis 24 hours before transplantation. Bilateral injections of stem cells or saline to subiculum were performed using a stereotaxic apparatus (Leica Biosystems, Buffalo Grove, MO, http://www.leicabiosystems.com) and the following coordinates relative to the bregma were used after adaption from the brain atlas by Paxinos and Watson: anteroposterior, 4.16; mediolateral, 3.25; dorsoventral, 4.00. Mice were anesthetized with a mixture of isoflurane gas and oxygen delivered via a nose cone. Mice were placed in the stereotaxic frame and injected with 100,000 stem cells or saline per side (4 μl per injection) using a Hamilton microsyringe (26‐gauge) and at an injection rate of 0.5 μl/min. Animal treatment and maintenance were approved by the Ethics Review Committee for Animal Experimentation of Seoul National University.

### Behavioral Analysis

Behavioral tests were conducted 2 months after injection. Spontaneous alteration performance in the Y‐maze was used to test the spatial working memory of the mice. The test was performed as described previously 25. Contextual fear‐conditioning testing was carried out in a fear‐conditioning chamber (H10‐11M‐TC, Coulbourn Instruments, Whitehall, PA, http://www.coulbourn.com/) fitted with metal shock grid. The test was conducted over 3 days. During the habituation period, cages containing the mice were kept in the testing room for 3 hours. During the training session, each mouse was allowed to explore the conditioning chamber for 4 minutes 30 seconds and received two electric foot shocks (0.65 mA, 2 seconds). For the context session on day 3, mice were placed in the chamber for 5 minutes, during which time no shock was applied. Time spent freezing was recorded with a chamber‐mounted camera. Freezing time was analyzed by FreezeFrame software (Coulbourn Instruments) connecting the conditioning chamber.

### Immunohistochemistry

For immunohistochemistry, male animals were sacrificed at 6 months of age. Mice were anesthetized with a Zoletil (Virbac, Carros, France, https://www.virbac.com) and Rompun (Bayer Korea, Seoul, Republic of Korea, https://www.bayer.co.kr/) cocktail solution (3:1 ratio, 1 ml/kg i.p.) and transcardially perfused with 4% paraformaldehyde (Biosesang, Seongnam, Republic of Korea, http://www.biosesang.com/). Serial 30‐mm‐thick coronal brain tissue sections were cut by using a freezing microtome (Leica Camera, Wetzlar, Germany, https://us.leica-camera.com/). To investigate neuronal loss in 5XFAD mice, tissue sections were pretreated with 1% hydrogen peroxidase and then incubated with mouse anti‐NeuN (1:1,000; EMD Millipore) at 4°C overnight. Subsequently, sections were incubated with biotinylated horse anti‐mouse IgG antibody (1:200; Vector Laboratories, Burlingame, CA, https://vectorlabs.com/) and avidin‐biotin‐peroxidase complex solution and then visualized with 3,3′‐diaminobenzidine as the chromogen. To detect immunofluorescence of molecules, tissue sections were incubated overnight at 4°C with the following the primary antibodies: biotin‐labeled 4G8 (1:2,000; Covance, Princeton, NJ, http://www.covance.com/), anti‐NeuN (1:2000; EMD Millipore), anti‐glial fibrillary acidic protein (GFAP; 1:2,000; Thermo Fisher), anti‐Iba‐1 (1:500; Wako, Osaka, Japan, http://www.wako-chem.co.jp), and anti‐Olig2 (1:1,000; EMD Millipore). For detecting Aβ, tissue sections were pretreated with 70% formic acid before incubation in biotin‐labeled 4G8 antibody. After washes in phosphate‐buffered saline, the sections were incubated with the following secondary antibodies (all obtained from Thermo Fisher): Alexa Fluor 488‐conjugated streptavidin (1:500), goat anti‐rabbit Alexa 594 (1:500), goat anti‐rat Alexa 488 (1:500), donkey anti‐mouse Alexa 647 (1:500) for 1.5 hours. All sections were imaged using a confocal laser scanning microscope (FluoView FV 10i, Olympus, Waltham, MA, http://www.olympus-lifescience.com)

### Quantification of Immunoreactivity

For quantification of NeuN‐, GFAP‐, Iba‐1‐, and Olig2‐positive cells, three sections (100 mm apart) from each mouse were taken from similar regions. Immunofluorescence images of the subiculum were taken by using a confocal laser scanning microscope (FluoView FV 10i). Stained cells were counted by using Image‐Pro Plus software, version 6.0 (Media Cybernetics, Bethesda, MD, http://www.mediacy.com/). To analyze biotin‐4G8‐positive areas, immunofluorescence‐positive regions in the subiculum were analyzed by using Fiji software (National Institutes of Health, Bethesda, MD, https://fiji.sc/). Maturation of oligodendrocytes was quantified for morphological assessment of dendritic branching as previously described 26.

### **Measurement of β or γ**‐**Secretase Activity**

In vitro peptide cleavage assay was performed as described for the measurement of β‐ or γ‐secretases activity 27.

### Tandem Liquid Chromatography/Mass Spectrometry Analysis

Subiculum samples were homogenized in ice‐cold hypotonic buffer (6 mM Tris‐HCl, 1 mM EDTA; pH, 7.8) containing protease inhibitor, phosphatase inhibitor, and phenylmethane sulfonyl fluoride by using a tissue grinder. The supernatant was obtained by centrifugation at 12,000 *g* for 10 minutes. The pellet was mixed with 200 μl of the supernatant in 0.5% SDS. The sample was ultrasonicated three times each, five per set; bicinchoninic acid (BCA) quantification was performed by using Micro BCA Protein Assay Kit (Thermo Fisher). Protein samples were digested with lys‐C and trypsin as follows. Briefly, 45 μl of 500 mM ammonium bicarbonate was added to 300‐μg aliquots of protein sample, and the final volume was adjusted to 100 μl with 8 M urea solution. A total of 5 μl of 200 mM dithiothreitol was added, and the resulting mixture was incubated for 1 hour; then, 5 μl of 300 mM iodoacetamide was added and the mixture was incubated for 30 minutes at room temperature in the dark. After incubation, the peptide mixtures were diluted to 1:10 with 50 mM ammonium bicarbonate, and lys‐C (Wako) solution was added. After overnight incubation at 37°C, trypsin was added (1:100; Promega). Trypsin digestion took place at 37°C for additional overnight incubation. The resulting peptides were purified by using Mixed‐Mode Cation‐eXchange (MCX) cartridge (Waters Corp., Milford, MA, http://www.waters.com) according to the manufacturer's instructions. The peptide mixtures were concentrated to near‐dryness using SpeedVac (Thermo Fisher), at which point peptide concentration was measured by using a Micro BCA Protein Assay before labeling with isobaric tags for relative and absolute quantitation (iTRAQ).

### Protein Digestion

Equal amounts of peptides (100 μg) were labeled by using the iTRAQ Reagents Multiplex Kit (Thermo Fisher). Dried peptide sample was resuspended in 20 μl of dissolution buffer consisting of triethylammonium bicarbonate (TEAB; pH, 9) and labeled individually with 114, 115, 116 and 117 iTRAQ reagents, which were reconstituted with 70 μl of ethanol at room temperature for 1 hour. The labeling reaction was stopped by drying in a SpeedVac. Obtained brown pellets were combined and cleaned by using Oasis MCX cartridge (Waters Corp., ). Four labeled peptide aliquots were combined and fractionated by high‐pH reverse‐phase chromatography as follows: A Sep‐Pak column (1 ml, Waters Corp.) was activated with MeOH and 50 mM TEAB in 80% acetonitrile (ACN) and then was equilibrated with TEAB. The combined iTRAQ‐labeled peptide samples were loaded onto the column and eluted with 50 mM TEAB in ACN (10%, 15%, 20%, 25%, 30%, 35%, 40%, 80% ACN). The eluted samples were then dried by using the CentriVap apparatus (Labconco, Kansas City, MO, http://www.labconco.com).

### Tandem Liquid Chromatography/Mass Spectrometry Analysis on Q‐Exactive Instrument

Peptides were resuspended in 30 μl of solvent A (0.1% formic acid in water), and 1 μl of sample was loaded onto a trap 75 μm (inner diameter microcapillary) × 2 cm C18 column (Thermo Fisher) and a Easy‐Spray 75 μm × 50 cm C18 column (Thermo Fisher) and separated with a gradient of 3%–5%–35% solvent B (0.1% formic acid in ACN) for 180 minutes at a flow rate of 250 nl/min. Mass spectrometry (MS) spectra were recorded on a Q‐Exactive (Thermo Fisher) hybrid quadrupole‐Orbitrap mass spectrometer interfaced with a nano‐ultra‐performance liquid chromatography (LC) system (Easy nLC 1000, Thermo Fisher). Standard MS condition of the spray voltage was set to 2.0 kV, and the temperature of the heated capillary was set to 250°C. Full scans were acquired in the mass analyzer at approximately 300–1600 m/z, with resolution of 70,000 for the full MS scans, normalized collision energy set to 32, and a resolution of 17,500 for high‐energy collisional dissociation fragmentation. The Q‐Exactive instrument was operated in data‐dependent mode, with one survey MS scan followed by 10 tandem MS (MS/MS) scans and a dynamic exclusion time of 20 seconds.

### Target‐Decoy Database Search

Monoisotopic masses of precursor ions in LC‐MS/MS data were refined by using post‐experiment monoisotopic mass refinement software before a database search 28. The resultant MS/MS data were searched against a composite target‐decoy database containing a mouse database (UniProt Release 2014_04; 51,597 entries; Uniprot, http://www.uniprot.org/) and its reversed complements using MS‐generating function (MS‐GF) + v9387 29. The searches were performed allowing for semitryptic peptides, and the maximum number of missed cleavage was set to 3. Mass tolerances of 10 ppm and 50 milli‐mass units were used for precursor and fragment ions, respectively. Fixed modification parameters at iTRAQ4Plex (N‐term), iTRAQ4Plex (K), and carbamidomethylation of cysteine (57.021460 Da) were used. Variable modification options were used for the oxidation of methionine (15.994920 Da), and the carbamylation of the N‐terminal site (43.005810 Da). For MS‐GF+ outputs, we used ComputeFDR ( https://bix-lab.ucsd.edu/display/CCMStools/ComputeFDR) to calculate false discovery rates (FDRs) for peptide‐spectrum matches (PSMs), and then selected PSMs with FDR < 0.01 as previously described 30.

### Identification of Differentially Expressed Proteins

The intensities of the multiplexed iTRAQ reporter ions from the triplicate experiments were normalized by using the quantile normalization method 31. Through use of the normalized intensities of the iTRAQ reporter ions, protein‐iPSC/vehicle ratios of the peptides at 1 day (116:114) and 2 months (117:115) were calculated in each replicate. With the peptide ratios in each replicate, the relative abundances (protein‐iPSC/vehicle ratios) of the proteins at 1 day or 2 months were calculated by using the linear‐programming method previously reported 32. To identify differentially expressed proteins (DEPs), a one‐sample Student's *t* test was applied to the relative protein abundances from three replicates at 1 day or 2 months (1 day_DEP or 2 month_DEP, respectively). The DEPs were then identified as those with at least two nonredundant peptides (*p* < .1 following the *t* test) and fold‐changes above the 90th percentiles in the fold‐change distribution (1.22‐fold for 1 day and 1.45‐fold for 2 months). Among the DEPs at 2 months, we further selected the proteins (2_month/1 day_DEP) whose changes at 2 months were significantly greater than those at 1 day. To this end, we applied a two‐sample Student's *t* test to the fold‐changes from 1 day and at 2 months. These fold changes were then normalized by dividing fold‐changes at 2 months by fold‐changes at 1 day. We selected a subset of the DEPs at 2 months that differed at *p* < .1 and that showed normalized fold‐changes >1.48 (90th percentile in the normalized fold‐change distribution).

### Enrichment Analysis of Gene Ontology Biological Processes

To identify cellular processes significantly represented by the DEPs (1 day_DEP, 2 month_DEP, or 2 month/1 day_DEP), we performed enrichment analysis of the gene ontology biological processes (GOBPs) for the DEPs using the ConsensusPathDB (CPDB) software 33. We then selected the GOBPs and Kyoto Encyclopedia of Genes and Genomes (KEGG) pathways significantly represented by the DEPs as those with *p* < .1 from hypergeometric test provided by the CPDB software and with ≥ 3 DEPs.

### Estimation of Cell Types Showing DEPs

To examine which types of cells exhibited differential expression indicated by DEPs at 1 day or 2 months, we assayed previously reported genes specifically expressed in astrocytes, oligodendrocytes, and neurons 34. Hypergeometric tests were performed to calculate *p* values representing the extent to which astrocyte‐, oligodendrocyte‐, and neuron‐specific genes were enriched in 1 day_DEPs, 2 month_DEPs, and 2 month/1 day_DEPs.

### In Vitro Differentiation of Protein‐iPSCs Into Oligodendrocytes

For oligodendrocyte differentiation, protein‐iPSCs were trypsinized (0.05%) and resuspended with embryoid body (EB) media on a culture dish for 30 minutes to eliminate feeder cells. EB formation was performed by the hanging drop method. A total of 1 × 10^4^ cells were contained in 20 μl of a droplet on the lid of a Petri dish. The next day, EBs were plated on 6‐well plate coated with Ultra‐Low Attachment Surface (Corning, Corning, NY, https://www.corning.com) for 10 days in NeuroCult Differentiation Kit medium (Stem Cell Technologies, Vancouver, British Columbia, Canada, https://www.stemcell.com). Four to five EBs were then attached to a polyornithine/fibronectin‐coated 35‐mm dish (ibidi, Planegg/Martinsried, Germany, http://ibidi.com/) for another 10 days. To evaluate the effect of transferrin, we cultured neuronal precursor cells in two conditions: One group was cultured in N2 media (DMEM/F‐12 media containing apotransferrin [100 μg/ml], insulin [25 μg/ml], sodium selenite [30 nM], progesterone [20 nM], putrescine [100 μM], penicillin [100 units/ml], and streptomycin [100 μg/ml]), and the other group was cultured in the absence of apotransferrin until the end of differentiation. For 4–6 days, neuronal precursor cells were grown in N2 supplemented with basic fibroblast growth factor 2 (20 ng/l) (R&D Systems) and platelet‐derived growth factor AA (10 ng/ml). To facilitate differentiation into mature oligodendrocytes, we cultured cells in N2 supplemented with T3 (3,3,5‐triiodo‐L‐thyronine sodium salt).

## Results

### Characterization of Mouse Protein‐iPSCs

**Figure 1:**
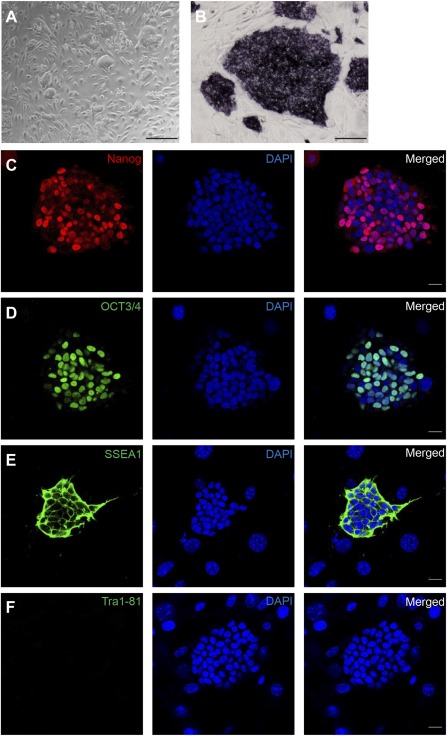
Characterization of mouse protein‐iPSCs. **(A):** Phase contrast morphology of protein‐induced pluripotent stem cell (iPSC) colonies. Scale bar = 500 μm. **(B):** Alkaline phosphatase staining of protein‐iPSC colonies. Scale bar = 200 μm. **(C–F):** Immunocytochemical staining shows that protein‐iPSCs express Nanog **(C)**, Oct4 (**D**), SSEA1 **(E),** but not Tra1‐81 **(F)** (a known human embryonic stem cell marker). Scale bar = 20 μm. Abbreviations: DAPI, 4′,6‐diamidino‐2‐phenylindole; SSEA1, stage‐specific embryonic antigen 1.

The protein‐iPSCs were cultured by using the same culture protocol as previously reported 21 22 23 24. The colonies were similar to the authentic mouse ES cell and classic iPSC colonies in morphology on feeder layers. They showed a large nucleus, minimal cytoplasm, and nondistinct cytoplasmic membranes between the cells (Fig. 1A). These colonies were expanded with subculture every 3–5 days. To confirm the stemness of our protein‐iPSCs, we carried out alkaline phosphatase (ALP) staining and immunocytochemistry. Protein‐iPSCs exhibited strong ALP activity (Fig. 1B) and expressed pluripotency markers, including Nanog, Oct4, and SSEA1 (Fig. 1C–1E). However, human stemness marker Tra1‐81 was not detected in these protein‐iPSCs (Fig. 1F). These data confirmed that protein‐iPSCs had been maintained homogeneously as pluripotent states.

### Engrafted Protein‐iPSCs Differentiated Into Glial Cells

**Figure 2:**
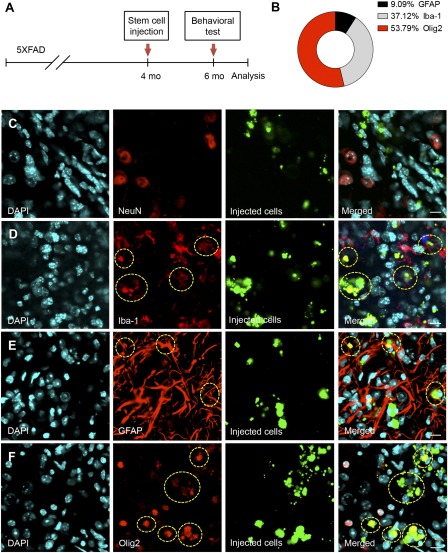
Protein‐induced pluripotent stem cells (iPSCs) differentiated into glial cells in the subiculum of 5XFAD mice. **(A):** Stereotaxic surgery was performed for injection of stem cells or saline. Two months after surgery, spatial learning and memory were assessed via Y‐maze and contextual fear conditioning test. Mice were sacrificed and brain sections analyzed by immunohistochemistry. **(B):** Confocal microscopy was used to investigate the fate of transplanted protein‐iPSCs, and quantitative analysis was performed. **(C):** Fluorescent nanoparticle‐tagged protein‐iPSCs were not colabeled with NeuN‐positive neurons but were found to surround other subiculum area. Scale bar = 10 μm. **(D–F):** Green fluorescent protein‐positive protein‐iPSCs colabeled with Iba‐1 (37.12%, **D**), GFAP (9.09%, **E**), and Olig2 (53.79%, **F**) (circles indicate colabeled cells). Scale bars = 10 μm. Abbreviations: DAPI, 4′,6‐diamidino‐2‐phenylindole; GFAP, glial fibrillary acidic protein.

To determine whether protein‐iPSCs ameliorate AD pathogenesis in 5XFAD mice, stem cell‐injected 5XFAD mice and their control and WT littermates were examined 2 months after stereotaxic injection in the subiculum area, where Aβ plaques are deposited at this age (Fig. 2A). After the behavioral test, mice were sacrificed and brains were evaluated for the fate of engrafted protein‐iPSCs. Immunohistochemical analysis demonstrated that engrafted protein‐iPSCs had differentiated into three lineages: microglia, astrocytes, and oligodendrocytes. Fluorescence staining of tissue sections revealed that most protein‐iPSCs differentiated into glial cells, with 9.09% of injected cells (nanoparticle tagged: green) coexpressing the astrocytic marker GFAP, 37.12% coexpressing the microglial marker Iba‐1, and approximately 53.79% coexpressing the oligodendrocytic marker Olig2 (Fig. 2B, 2D, 2F). Interestingly, we did not observe any proportion of protein‐iPSCs adopting a neuronal fate, signified by coexpression with the neuronal marker NeuN (Fig. 2C). These data indicate that the subiculum niche promoted the differentiation of protein‐iPSCs into glial cells, not neurons.

### Protein‐iPSCs Reduced Plaque Deposition but Did Not Ameliorate Neuronal Loss

**Figure 3:**
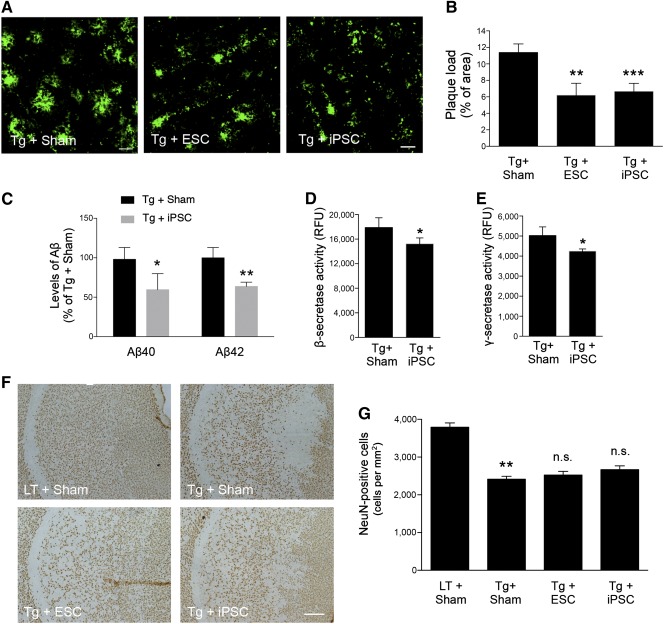
Effect of transplanted stem cells to Alzheimer's disease pathology. **(A):** Coronal brain sections were stained with biotin‐4G8 antibody to detect Aβ deposition after saline or stem cell transplantation. Scale bars = 20 μm. **(B):** Bar graph shows quantification of 4G8‐positive area (*n* = 10 per group). ∗∗, *p* < .01; ∗∗∗, *p* < .001. **(C):** Levels of formic acid‐soluble Aβ 40 and Aβ 42 measured by using an enzyme‐linked immunosorbent assay kit for human‐specific Aβ level in brain homogenates from the protein‐iPSC‐injected 5XFAD mice versus the saline‐treated 5XFAD mice (*n* = 5 per group). ∗, *p* < .05; ∗∗, *p* < .01. **(D, E):** β‐secretase **(D)** and γ‐secretase **(E)** activities were measured by using an in vitro peptide cleavage assay (*n* = 5 per group). ∗, *p* < .05. **(F):** Representative images of NeuN‐positive neurons. Scale bar = 200 μm. **(G):** Bar graph shows quantification of NeuN‐positive cells (*n* = 10 per group). Error bars represent the mean ± SEM. ∗∗, *p* < .01. Abbreviations: ESC, embryonic stem cell; iPSC, induced pluripotent stem cell; LT, littermate; RFU, relative fluorescent unit.

To determine whether injection of stem cells modulates AD pathology, we examined brain changes using immunohistochemical and histological analysis. We examined plaque deposition in the subiculum by measuring the immunofluorescent density of the 4G8 (Aβ‐specific monoclonal antibody)‐positive signal and found the deposition of Aβ to be markedly reduced following protein‐iPSCs transplantation in the 5XFAD mice as well as in the ESC‐transplanted mice as a control compared with that in the sham‐injected mice (Fig. 3A, 3B). In addition, the brains of protein‐iPSC‐injected 5XFAD mice showed significantly reduced levels of Aβ40 and Aβ42 compared with saline‐injected 5XFAD mice (Fig. 3C). We investigated whether protein‐iPSCs influenced the pathway of Aβ generation. To measure β‐ and γ‐secretase activities, we performed an in vitro peptide cleavage assay in the brains of protein‐iPSC‐injected 5XFAD mice. Protein‐iPSC‐injected mice showed significant attenuation of both β‐ and γ‐secretase activities compared with saline‐injected 5XFAD mice (Fig. 3D, 3E). On the other hand, Western blot analysis showed no difference in expression of full‐length APP, a disintegrin and metallopeptidase domain 10 (ADAM10) (α‐secretase), BACE1 (β‐secretase), or presenilin enhancer 2 (PEN2, γ‐secretase component) ( supplemental online Fig. 1). This implies that the brains of protein‐iPSC‐injected mice are subject to decreased activities of both β‐ and γ‐secretases without changes in secretase‐related protein expressions. To test whether transplantation of stem cells influences progressive neuronal loss, quantitative analysis of the number of neurons in the subiculum was performed. As expected, a significant decrease in the number of NeuN‐positive neurons within the subiculum was observed in 5XFAD mice compared with littermates (Fig. 3G). However, quantitative analysis revealed no significant neuronal recovery in the stem cell‐injected 5XFAD mice versus age‐matched saline‐injected 5XFAD mice (Fig. 3F, 3G). These findings imply that engrafted protein‐iPSCs do not differentiate into neurons and that other bystander effects of stem cells may ameliorate Aβ plaque deposition through modulation of both β‐ and γ‐secretase activities of 5XFAD.

### Protein‐iPSC Transplantation Led to Proteome Changes in the Subiculum of 5XFAD Mice

**Figure 4:**
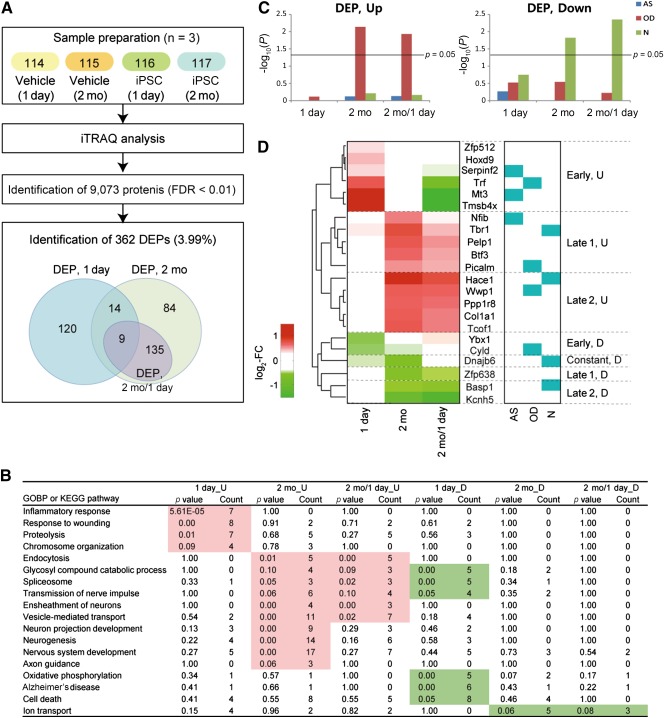
Identification of differentially expressed proteins and integration of neural cell type specific genes. **(A):** Overview of global proteome analysis of subiculum tissues obtained 1 day and 2 months after vehicle and protein‐iPSC transplantation. **(B):** GOBPs and KEGG pathways represented by 1 day_DEPs, 2 month_DEPs, and 2 months/1 day_DEPs. For each GOBP or KEGG pathway, the table provides the enrichment *p* value and the number of DEPs (count) that belong to the GOBP or KEGG pathway. The GOBP or KEGG pathways significantly (*p* ≤ 0.1 and count ≤ 3) enriched by the DEPs in each condition are highlighted in background (red and green for the enrichment by up‐ and downregulated DEPs, respectively). **(C):** Bar graphs showing the significance of the overlapping DEPs with N‐, AS‐, and OD‐specific genes previously reported. The heights of each bar graph represent –log10(P), where the *p* value represents the significance of the overlaps between the DEPs and cell‐specific genes. The horizontal line indicates *p* = .05. **(D):** Heat map showing differential expression of the 20 DEPs with transcriptional regulation activities at 1 day and 2 months. Red and green represent up‐ and downregulation, respectively, in protein‐iPSC‐transplanted samples, compared with vehicle‐injected samples. The color bar shows the gradient of log‐two‐fold‐change between protein‐iPSC‐ and vehicle‐transplanted samples. The right heat map indicates whether each DEP belongs to the genes specific to N, AS, and OD. Abbreviations: AS, astrocyte; D, downregulated; DEP, differentially expressed proteins; FDR, false discovery rate; GOBP, gene ontology biological processes; iTRAQ, isobaric tags for relative and absolute quantitation; KEGG, Kyoto Encyclopedia of Genes and Genomes; N, neuron; OD, oligodendrocyte; U, upregulated.

To understand the effect of protein‐iPSC transplantation on AD pathology and differentiation of transplanted protein‐iPSCs at the molecular level, we performed global proteome profiling of subiculum tissues obtained from 5XFAD mice after transplantation of protein‐iPSCs (1 day and 2 months later). For quantitative proteome analysis, we labeled the following subiculum samples using iTRAQ agents (Fig. 4A): control samples collected 1 day and 2 months after vehicle transplantation (114 and 115, respectively) and samples collected 1 day and 2 months after protein‐iPSCs transplantation (116 and 117, respectively). We collected the sample 1 day after injection to avoid injection effects and to compare before‐and‐after differentiation of protein‐iPSCs at the subiculum of 5XFAD mice. The labeled samples were fractionated into eight fractions by using a previously reported high‐pH fractionation method 35, and LC‐MS/MS analysis was then performed for each fraction three times by using a Q Executive mass spectrometer (Fig. 4A). With the target‐decoy database search using MS‐GF+ (see Materials and Methods for details), we identified 9,073 proteins with an FDR of <0.01 (the proportion of false discoveries among whole discoveries). Among them, we selected 362 DEPs between protein‐iPSC‐ and saline‐transplanted samples (143 were 1 day_DEPs and 242 were 2 month_DEPs) as described in Materials and Methods (Fig. 4A; supplemental online Table 1). Among 2 month_DEPs, we further selected 144 DEPs (2 month/1 day_DEPs) whose changes were significantly greater than those at 1 day, thus representing the proteome affected when protein‐iPSCs are fully differentiated.

To understand the cellular processes represented by these DEPs, we performed enrichment analysis of GOBPs and KEGG pathways using CPDB software 33. Stem cell‐related proteins (adenomatous polyposis coli protein; ribosomal protein L family) were significantly (*p* < .1) represented by upregulated proteins, among 1 day_DEPs, in protein‐iPSC‐transplanted samples, compared with controls ( supplemental online Table 1). On the other hand, AD‐related KEGG pathways (oxidative phosphorylation and Alzheimer's disease) were significantly represented by downregulated proteins among 1 day_DEPs (Fig. 4B). Notably, neurodegeneration‐related GOBPs (ensheathment of neurons, transmission of nerve impulse, and vesicle‐mediated transport) were represented by mostly upregulated proteins among 2 month_DEPs (Fig. 4B). This is consistent with our observations that behaviors of 5XFAD mice after iPSC transplantation were improved.

### Oligodendrocyte‐Specific Genes Are Significantly Enriched in Upregulated DEPs

To examine which cell types reflect the changes in the subiculum proteome after protein‐iPSC transplantation, we integrated the DEPs with neural cell‐type markers and the previously reported genes specifically expressed in neurons, astrocytes, and oligodendrocytes (1,441, 1,489, and 1,371 genes, respectively) 34. The upregulated proteins showed significant overlap with the oligodendrocyte‐specific genes (Fig. 4C, left panel). In contrast, the downregulated proteins showed significant overlap with the genes specific to neurons (2 months, 2 month/1 day) (Fig. 4C, right panel). These data indicate that oligodendrocyte‐specific features were increased 2 months after protein‐iPSC transplantation. The latter is consistent with the finding that ensheathment of neurons was significantly represented by the 2 month_DEPs (Fig. 4B). Notably, neuron‐specific features were decreased constantly throughout the transplantation. Taken together, these data suggest that the transplanted protein‐iPSCs differentiate into oligodendrocytes 2 months after transplantation.

### Transferrin Can Promote Differentiation of Protein‐iPSCs Into Oligodendrocytes

**Figure 5:**
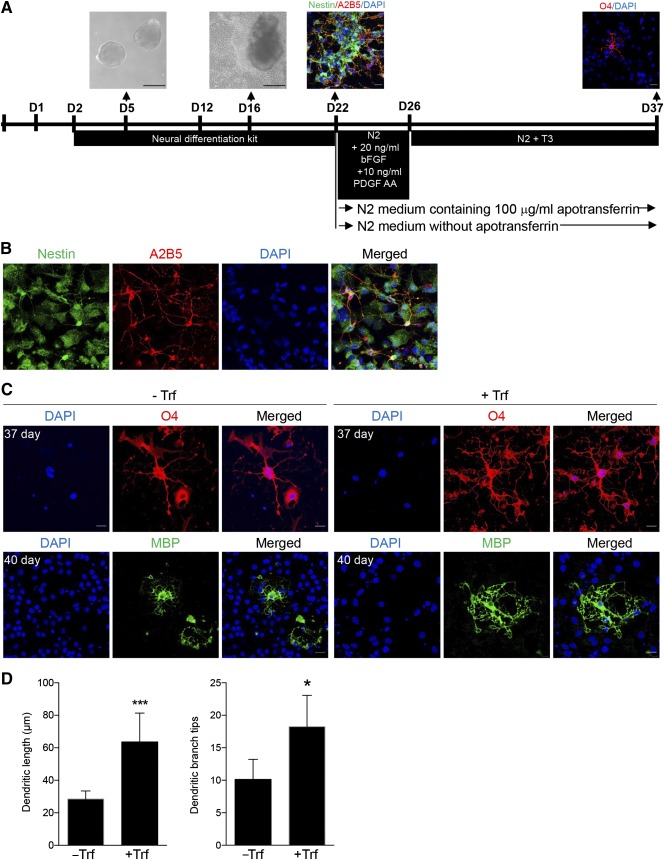
Transferrin promotes differentiation of protein‐induced pluripotent stem cells (iPSCs) into oligodendrocytes. **(A):** Timeline of differentiation of protein iPSCs into oligodendrocytes. Medium was replaced every other day. After day 22, transferrin was withdrawn from the medium until the end of differentiation. Phage‐contrast image: scale bar = 200 μm. Fluorescent image: scale bar = 20 μm. **(B):** Protein‐iPSC‐derived oligoprecursor cells were stained on day 22 with anti‐Nestin and anti‐A2B5. Nuclei were stained with DAPI. Scale bar = 20 μm. **(C):** Immunofluorescence images of differentiated oligodendrocytes in two different differentiation conditions. Oligodendrocytes differentiated in the absence of (−Trf) and in the presence of apotransferrin (+Trf). O4 was stained after 37 days of differentiation. Myelin basic protein‐positive cells were visualized by immunofluorescence staining 40 days after differentiation. Scale bar = 20 μm. **(D):** Quantification of the length and number of dendritic branches. ∗, *p* < .05; ∗∗∗, *p* < .001 (*n* = 8). Abbreviations: DAPI, 4′,6‐diamidino‐2‐phenylindole; MBP, myelin basic protein; PDGF, platelet‐derived growth factor; Trf, apotransferrin.

Differentiation of transplanted protein‐iPSCs into glial cells might be important in recovering neurological functions. For example, differentiation of the protein‐iPSCs into oligodendrocytes may ameliorate certain functions, such as ensheathment of axons or transmission of nerve impulse, that are degenerated in AD conditions 36, as indicated by the GOBP enrichment analysis (Fig. 4B). Thus, we examined which regulators can promote differentiation of protein‐iPSCs into glial cells. To this end, we focused on 22 DEPs that can act as regulators of transcription based on gene ontology biological processes and transcription factor databases 37 38 39 (Fig. 4D). Among the 22 DEPs, 6 (Zfp512, Hoxd9, Serpinf2, apotransferrin [Trf], Mt3, and Tmsb4x) were upregulated at 1 day. Of the 6, Trf, the iron‐free form of transferrin, added to cultured oligodendrocyte progenitor cells, inhibited their migration and then enhanced their differentiation into oligodendrocytes 40. However, a role for transferrin in differentiation of protein‐iPSCs into glial cells has not been reported. Thus, we hypothesized that early upregulation of Trf after protein‐iPSC transplantation can promote differentiation of protein‐iPSCs into oligodendrocytes. To test this hypothesis, we differentiated protein‐iPSCs into oligodendrocytes in vitro, as described in Figure 5A. Nestin and A2B5‐positive cells were extended from attached EBs (Fig. 5B). To identify whether transferrin promoted differentiation of protein‐iPSCs into oligodendrocytes, two different conditions were used for neuronal precursor cells until mature oligodendrocyte appeared: Cells were cultured with or without apotransferrin after detection of A2B5 (oligodendrocyte precursor marker)‐positive cells. Markers of oligodendrocyte maturation (O4 and MBP) decreased significantly when cells were cultured in the absence of apotransferrin (Fig. 5C). To quantify the efficiency of maturation, we measured the length and number of dendritic branches. We found apotransferrin significantly promoted the maturation of oligodendrocyte differentiation (Fig. 5D).

### Stem Cell Transplantation Into Brain Improves Behavioral Impairments of 5XFAD Mice

**Figure 6:**
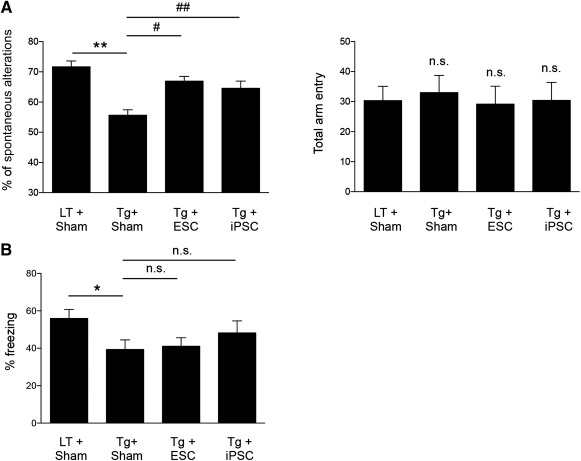
Stem cell transplantation improves cognitive impairment. **(A):** Cognitive function of the stem cell‐injected 5XFAD mice was assessed by using a Y‐maze task 2 months after surgery. The total number of arm entries did not differ between groups. In contrast, decreased spontaneous alteration was recovered in the stem cell‐injected 5XFAD mice compared with saline‐treated 5XFAD mice (*n* = 13 per group). ∗∗, *p* < .01 versus LT + sham; #, *p* < .05, ##, *p* < .01 versus Tg + Sham. **(B)**: Cognitive function of the stem cell‐injected 5XFAD mice was assessed by using a contextual fear conditioning task 2 months after surgery (*n* = 13 per group). Error bars represent the mean ± SEM. ∗, *p* < .05 versus LT + sham. Error bars represent the mean ± SEM. Abbreviations: ESC, embryonic stem cell; iPSC, induced pluripotent stem cell; LT, littermate; n.s., not significant; Tg, transgenic mice.

To determine whether transplanted stem cells could improve spatial learning and memory in an AD mouse model, we performed stereotaxic injections with 100,000 murine ESCs (as stem cell controls) or protein‐iPSCs to the subiculum regions of 5XFAD and age‐matched littermates. Two months after injection, stem cell‐transplanted 5XFAD mice, their controls (saline‐injected), and WT littermates were tested on the Y‐maze and on contextual fear conditioning (behavioral tests of learning and memory function) 2 months after injection (Fig. 6A). Saline‐injected 5XFAD mice showed significant memory alteration in the Y‐maze test compared with age‐matched saline‐injected littermates. However, injection of ESCs and protein‐iPSCs into 5XFAD mice significantly ameliorated cognitive impairments, as indicated by lower spontaneous alteration rates during Y‐maze test (Fig. 6A). Total arm entries were similar among all tested groups, indicating that memory deficits were recovered by the injection of both protein‐iPSCs and ESCs without changing mobility. In the contextual fear conditioning test, littermates showed normal freezing behavior following shock in a conditioned context. As expected, saline‐injected 5XFAD mice were impaired, freezing only approximately 40% of the time in the conditioned context (Fig. 6B). Although the finding was not statistically significant, protein‐iPSC‐injected mice showed a slight amelioration of this memory deficit (Fig. 6B). These results suggest that protein‐iPSC transplantation can help restore cognitive function in impaired 5XFAD mice.

## Discussion

Recently, Takahashi and Yamanaka have generated pluripotent stem cells from mouse fibroblast with four transcription factors (Oct3/4, Sox2, c‐Myc, and Klf4). These ES‐like cells are named iPSCs 23. iPSCs overcome many disadvantage of ESCs, including ethical controversy and immune rejection following transplantation in patients. Nonetheless, there are still considerations, such as cost, safety, and efficiency, in generating iPSCs. Therefore, to resolve safety issue, we developed a new method to generate iPSCs without any genetic modification but with only proteins 21. These protein‐derived iPSCs are converted from somatic to ES‐equivalent status in global gene expression and epigenetic status, such as DNA methylation and histone modifications. Furthermore, these ES equivalents are biologically and functionally indistinguishable from authentic ESCs.

In our study, we used 5XFAD mice that overexpress five familial AD transgenes to test the hypothesis that protein‐iPSCs have the capacity to ameliorate AD pathogenesis in vivo. Here we report that protein‐derived iPSC‐injection restores the cognitive impairment accompanying Aβ‐pathology in 5XFAD mice (Fig. 6A, 6B). Interestingly, 5XFAD mice treated with protein‐iPSCs exhibited decreased plaque deposition. Although we cannot show the results including the Tg+ESC group, this was due to mitigation of both β‐ and γ‐secretase activities, without restoration of neuronal cell loss (Fig. 3; supplemental online Fig. 1). The Tg+ESC group will be studied for precise mechanism of effect in AD mouse model as a further study. These data imply that the beneficial effects of protein‐iPSCs on cognition are not directly mediated by neuronal protection or restoration. Although Kim et al. reported that mesenchymal stem cells decreased β‐ and γ‐secretase activity, and other groups found that transplanted stem cells may attenuate Aβ generation 16, 17, the mechanism by which Aβ plaque deposition can be reduced without concomitant recovery of neurons by stem cells is unclear. Some researchers have reported that stem cell niche, or microenvironment, is crucial for supporting the activity and behavior of stem cells after transplantation 41, 42. On the basis of these observations, it is possible that niche‐derived extrinsic signals may influence Aβ generation. Various signaling pathways, including the AMP‐activated protein kinase (AMPK) pathway, regulate Aβ generation 43, 44. Several studies have reported that AMPK signaling activation ameliorates Aβ production via autophagosome formation that can facilitate degradation of Aβ 45, 46. We found that STE20‐related kinase adaptor α and protein tyrosine phosphatase 1, which can activate AMPK signaling according to KEGG pathway, were upregulated in our protein‐iPSC‐injected 5XFAD mice ( supplemental online Table 2) 47, 48. Although our data provide evidence for decreased plaque formation following injection of protein‐iPSCs, additional detailed studies are required to understand the precise mechanisms underlying the changes observed here.

iPSCs are pluripotent cells that can be differentiated into various cell types, including neurons, microglia, astrocytes, and oligodendrocytes 49, 50. In addition, several lines of evidence suggest that engrafted neural stem cells differentiated into a variety of cell types 51 52 53. To address this issue, we investigated the fate of transplanted protein‐iPSCs and found a considerable number of glial cells, including oligodendrocytes (Olig2+), microglia (Iba‐1+), and astrocytes (GFAP+), that were double‐labeled with various markers of iPSCs. In parallel with previous data, transplanted protein‐iPSCs did not differentiate into neuronal cells (Fig. 2C). Recent studies have reported impairment of oligodendrocytes in AD patients and in AD animal models. Oligodendrocytes may exhibit increased apoptosis in AD conditions, but the precise mechanisms controlling their degeneration have not been clearly elucidated 7, 54, 55. Glial cells, including oligodendrocytes, support neuronal energy metabolism by regulating energy supply. Lee et al. demonstrated that oligodendrocytes release lactate via MCT1, monocarboxylate transporter 1, to support metabolic shuttling between astrocytes and neurons 56. According to this work, newly differentiated healthy oligodendrocytes may improve memory function by maintaining axonal integrity in neuronal networks 57, 58. Consistent with this hypothesis, we found that most transplanted protein‐iPSCs differentiate into oligodendrocytes and that oligodendrocyte‐related genes, particularly transferrin, are highly expressed in protein‐iPSC‐injected brains (Fig. 4). It is possible that transferrin in the stem cell niche promotes differentiation of protein‐iPSCs into oligodendrocytes, allowing adjacent neurons to receive energy‐generating metabolites 59.

## Conclusion

Taken together, our results suggest a novel role for protein‐iPSCs in restoring cognition in AD via plaque reduction and oligodendrocyte‐derived neuronal support.

## Author Contributions

M.‐Y.C., and Y.‐W.K.: study design, performance of experiments, collection and/or assembly of data, manuscript writing, final approval of manuscript; H.‐S.A. and H.J.: study design, performance of experiments, manuscript writing, final approval of manuscript; Y.Y.L.: performance of LC‐MS/MS analysis, data interpretation, manuscript writing; M.M.: experiment design, assistance with experiments; S.H.B., D.K.K., and H.S.: technical assistance in animal surgery; E.C.Y., D.H.: experiment design, data analysis; H.‐S.K., and I.M.‐J.: project design, supervision, manuscript writing, final approval of manuscript.

## Disclosure of Potential Conflicts of Interest

The authors indicated no potential conflicts of interest.

## Supporting information

Supporting Information
