# Supplementary material for: Protein‐Induced Pluripotent Stem Cells Ameliorate Cognitive Dysfunction and Reduce Aβ Deposition in a Mouse Model of Alzheimer's Disease
Source: Stem Cells Transl Med. 2016 Aug 15;6(1):293–305. doi: 10.5966/sctm.2016-0081 (PMC5442740; doi:10.5966/sctm.2016-0081)
Supplement: Supplementary file 1 — Supporting Information [file SCT3-6-293-s001.pdf]

Supplemental Information – Jung and Kim et al.

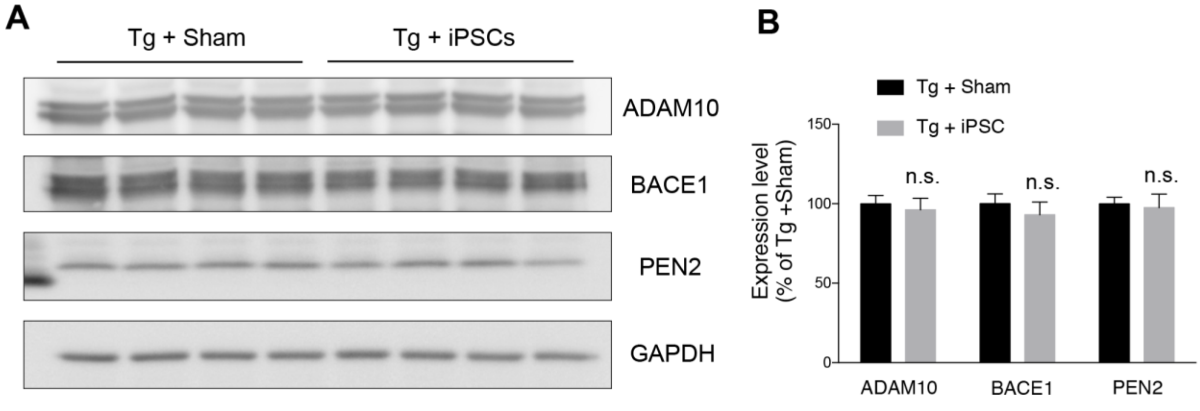

Supporting Information Fig. S1. APP processing in the brain of iPSCs-injected mice. (A) Representative immunoblot of α-, β- and γ-secretase expression levels in the brains of iPSCs-injected 5XFAD mice versus the saline-treated 5XFAD mice. (B) Densitometric quantification of immunoblot after normalization with GAPDH. n.s.: non-significant.

GeneID (mouse)	Symbol	GeneID (human)	SyStemCell (stem cell/control) Transcriptome	Proteome	DEP 1day P	log$_2$-FC	DEP	2mo P	log$_2$-FC	DEP
11657	Alb	213	1	1	0.00	0.59	U	0.07	-0.09	-
11789	Apc	324	1	1	0.06	0.32	U	0.30	0.16	-
19241	Tmsb4x	7114	1	1	0.02	2.56	U	0.12	-0.15	-
19944	Rpl29	6159	1	1	0.01	0.42	U	0.13	-0.06	-
20104	Rps6	6194	1	1	0.02	0.30	U	0.33	0.07	-
20639	Snrpb2	6629	1	1	0.02	0.29	U	0.32	NaN	-
22041	Trf	7018	1	1	0.02	0.75	U	0.86	0.02	-
56088	Psmg1	8624	1	1	0.06	0.31	U	0.50	0.14	-
67126	Atp5e	514	1	1	0.09	0.29	U	0.03	0.35	-
68436	Rpl34	NaN	1	1	0.04	0.83	U	0.06	0.50	-
76582	Ipo11	51194	1	1	0.08	0.41	U	0.76	-0.02	-
76808	Rpl18a	6142	1	1	0.01	0.34	U	0.39	0.04	-
114641	Rpl31	6160	1	1	0.00	0.38	U	0.01	0.43	-

Criteria of DEPs

- 1day : P < 0.1, FC>90th percentiles in fold change distribution (0.2869)
- 2mo : P < 0.1, FC>90th percentiles in fold change distribution (0.5361)

Supporting Information Table S1. Stem cell related proteins among 1 day Up-DEPs

Symbol	Description	1day			2mo		
		log2-FC	P-value	DEP	log2-FC	P-value	DEP
Strada	STE20-related kinase adaptor alpha	0.10	0.16	-	0.58	0.07	U
Ptpn1	protein tyrosine phosphatase, non-receptor type 1	0.29	0.01	U	0.17	0.93	-

Criteria of DEPs

- 1day : P < 0.1, FC>90th percentiles in fold change distribution (0.2869)
- 2mo : P < 0.1, FC>90th percentiles in fold change distribution (0.5361)

Supporting Information Table S2. Differential expression of AMPK signaling proteins
